# Deciphering the Systemic Impact of Herbal Medicines on Allergic Rhinitis: A Network Pharmacological Approach

**DOI:** 10.3390/life14050553

**Published:** 2024-04-25

**Authors:** Sa-Yoon Park, Yoon Yeol Lee, Min Hee Kim, Chang-Eop Kim

**Affiliations:** 1 Department of Physiology, College of Korean Medicine, Gachon University, Seongnam 13120, Republic of Korea; 2 Biomedical Research Institute, Seoul National University Hospital, Seoul 03080, Republic of Korea; 3 Department of Ophthalmology, Otolaryngology, and Dermatology, Kyung Hee University College of Korean Medicine, Kyung Hee University Hospital at Gangdong, Seoul 05278, Republic of Korea

**Keywords:** allergic rhinitis, traditional Asian medicine, herbal medicine, medicinal plants, network pharmacology, clustering analysis

## Abstract

Allergic rhinitis (AR) is a systemic allergic disease that has a considerable impact on patients’ quality of life. Current treatments include antihistamines and nasal steroids; however, their long-term use often causes undesirable side effects. In this context, traditional Asian medicine (TAM), with its multi-compound, multi-target herbal medicines (medicinal plants), offers a promising alternative. However, the complexity of these multi-compound traits poses challenges in understanding the overall mechanisms and efficacy of herbal medicines. Here, we demonstrate the efficacy and underlying mechanisms of these multi-compound herbal medicines specifically used for AR at a systemic level. We utilized a modified term frequency–inverse document frequency method to select AR-specific herbs and constructed an herb–compound–target network using reliable databases and computational methods, such as the Quantitative Estimate of Drug-likeness for compound filtering, STITCH database for compound-target interaction prediction (with a high confidence score threshold of 0.7), and DisGeNET and CTD databases for disease-gene association analysis. Through this network, we conducted AR-related targets and pathway analyses, as well as clustering analysis based on target-level information of the herbs. Gene ontology enrichment analysis was conducted using a protein–protein interaction network. Our research identified 14 AR-specific herbs and analyzed whether AR-specific herbs are highly related to previously known AR-related genes and pathways. AR-specific herbs were found to target several genes related to inflammation and AR pathogenesis, such as PTGS2, HRH1, and TBXA2R. Pathway analysis revealed that AR-specific herbs were associated with multiple AR-related pathways, including cytokine signaling, immune response, and allergic inflammation. Additionally, clustering analysis based on target similarity identified three distinct subgroups of AR-specific herbs, corroborated by a protein–protein interaction network. Group 1 herbs were associated with the regulation of inflammatory responses to antigenic stimuli, while Group 2 herbs were related to the detection of chemical stimuli involved in the sensory perception of bitter taste. Group 3 herbs were distinctly associated with antigen processing and presentation and NIK/NF-kappa B signaling. This study decodes the principles of TAM herbal configurations for AR using a network pharmacological approach, providing a holistic understanding of drug effects beyond specific pathways.

## 1. Introduction

Allergic rhinitis (AR) is an immunoglobulin E (IgE)-mediated inflammatory response of the nasal mucous membranes, with symptoms of rhinorrhea, nasal congestion, nasal itching, and sneezing [1]. The prevalence of AR is 10–30% worldwide and 16.1% in South Korea [2,3]. AR is not a life-threatening disease; however, it has a considerable impact on patients’ quality of life and contributes to worsening socioeconomic burden [4]. The total direct medical cost of AR was approximately USD 3.4 billion in the United states [5], and USD 223.68 million (direct and indirect USD 272.92 million) in South Korea [6]. Furthermore, AR is a systemic allergic disease that is generally associated with numerous multi-morbid disorders, including asthma, eczema, conjunctivitis, chronic middle ear effusions, rhinosinusitis, obstructive sleep apnea, and consequent behavioral and educational effects [7].

In many patients with AR, symptoms persist for years. Therefore, it is necessary to develop medicines with no adverse effects when employed as long-term therapy. Currently, the main medications used for AR include antihistamines, nasal steroids, nasal decongestants, and leukotriene receptor antagonists. However, long-term use of many AR medications can result in adverse effects. Therefore, traditional Asian medicine (TAM), which involves the use of medicinal herbs, has recently gained attention as a potential treatment for AR [8].

Unlike conventional drugs that contain a single active ingredient that acts on a single target, herbal medicines in TAM contain multi-compounds and are known to act on multi-targets simultaneously [9,10]. Therefore, identifying the overall mechanisms and efficacy of herbal medicines using conventional pharmacological analyses is challenging. Network pharmacology is an effective method for investigating the efficacy and mechanisms of multi-compounds at the system level, and has been widely applied to the exploration of candidate combinations of medicinal herbs for drug development and the prediction of possible mechanisms and adverse effects of herbal prescriptions [11,12]. To the best of our knowledge, little research has been conducted on the network pharmacological approach to systematically analyze the mechanisms of medicinal herbs used for treating AR.

In the present study, we aimed to investigate the potential effects and mechanisms of representative herbs used for treating AR in TAM, using a network pharmacological approach. We identified AR-specific herbs using a term frequency–inverse document frequency (TF-IDF) and investigated whether AR-specific herbs are highly related to previously known AR-related genes and pathways by applying a network pharmacological approach. Furthermore, we investigated the efficacy and mechanisms of TAM herbal prescriptions, including AR-specific herbs, by analyzing the protein–protein interaction (PPI) network of clustered groups of herbs.

## 2. Materials and Methods

### 2.1. Selection of AR-Related Herbal Prescriptions and AR-Specific Herbs

Among the numerous TAM herbal prescriptions used for AR, we selected seven from the AR Clinical Practice Guidelines for Korean Medicine published in 2021 [13], which are Socheongryong-tang, Okbyungpoong-san, Bojungikgi-tang, Mahwangbujaseshin-tang, Hyongeyonggyo-tang, Samsoeum, and Galgeun-tang. We selected five more prescriptions from the most commonly prescribed herbal formulas for AR in South Korea [14], which are Yeotaectonggi-tang, Younggamgangmisinhayin-tang, Insampaedok-san, Yukmijihwang-tang, and Maekmundong-tang. In this study, we defined the 12 herbal prescriptions as representative prescriptions for AR (Table S1).

For the selection of AR-specific herbs from representative prescriptions, we used the TF-IDF. The TF-IDF is a numerical statistic that reflects how important a word is to a document in a collection. In the current context, a word corresponds to an herb, and a document corresponds to a prescription. Term frequency, $tf\left(h,R\right)$, is the relative frequency of the herb h within the representative prescription R.

$$tf\left(h,R\right)=\frac{f_{h,R}}{\sum_{h^{\prime }\in R}f_{h^{\prime },R}}$$

where $f_{h,R}$ is the raw count of the herb h in the prescription R. The denominator is simply the total number of herbs in the representative prescription R.

The inverse document frequency, $idf\left(h,P\right)$ is a measure of how much information the herb provides, that is, whether it is common or rare across all herbal prescriptions.

$$idf\left(h,P\right)=\ln\frac{\left|P\right|}{\left|\left\{p\in P:h\in p\right\}\right|}$$

where |P| is the total number of prescriptions in the TCMID, a TAM database. The denominator $\left|\left\{p\in P:h\in p\right\}\right|$ is the number of prescriptions that include the herb h.

The TF-IDF is calculated as:

$$tfidf\left(h,R,P\right)=tf(h,R)\times idf(h,P)\times 100$$

The higher the $tfidf\left(h,R,P\right)$ value for a specific herb, it is considered to be an herb that is used more specifically for AR. Herbs with the $tfidf\left(h,R,P\right)$ value > 10 were selected as AR-specific herbs (Table S2).

Selected 14 AR-specific herbs are as follows: ephedra intermedia Schrenk and C.A.Mey., pueraria montana var. lobata (Willd.) Maesen & S.M.Almeida ex Sanjappa & Predeep, wolfiporia extensa (Peck) Ginns, pinellia ternata (Thunb.) Makino, glycyrrhiza glabra L., asiasarum sieboldii Miq., cinnamomum cassia (L.) J.Presl, bupleurum falcatum L., platycodon grandiflorum A.DC., astragalus mongholicus Bunge, angelica decursiva Franch. & Sav., aralia continentalis Kitag, panax ginseng C.A.Mey., and Saposhnikovia divaricata (Turcz.) Schischk. The plant name has been checked with “World Flora Online” (www.worldfloraonline.org (accessed on 1 June 2023)).

### 2.2. Disease-Related Targets and Pathway Analysis

To estimate the extent to which the potential targets of AR-specific herbs overlap with known AR genes, AR-related genes were retrieved from the Comparative Toxicogenomics Database (CTD; http://ctdbase.org/about/ (accessed on 1 June 2023)) and DisGeNET [15]. The CTD is a publicly available database that provides manually curated information, including chemical-disease and gene-disease relationships. The retrieved genes were restricted to those with direct evidence, that is, a curated association with AR. DisGeNET contains one of the largest publicly available collections of genes and variants associated with human disease. In DisGeNET, genes with a gene-disease association score > 0.3 were retrieved, and the threshold was selected according to the elbow method [16]. AR-related genes that satisfied these two conditions were analyzed in this study.

To infer the relations between potential targets of AR-specific herbs and AR-associated pathways, gene set enrichment analysis (GSEA) based on Kyoto Encyclopedia of Genes and Genomes (KEGG) pathway database [17] and manually curated pathways from AR studies was conducted. In GSEA, adjusted *p*-values and combined scores, the logarithm of the multiplication of the *p*-value and z-score of overlap between targets and gene sets were calculated. All enrichment analyses were conducted using Enrichr, an open-source, freely available web-based enrichment analysis tool [18]. We defined a combined score of an herb as the sum of the combined scores of its compounds with adjusted *p*-values lower than 0.05.

### 2.3. Construction of an Herb–Compound–Target Network

The TCM-mesh database [19] was used to obtain information regarding the compounds in AR-specific herbs. Compounds that may not affect oral administration were filtered out based on the quantitative estimate of drug-likeness (QED), i.e., a drug-likeness score based on molecular descriptors ranging from 0 to 1 [20]. Since a value of 0.35 is the mean QED for oral drugs approved by the Food and Drug Administration, we used it as the cut-off value for the compounds [21]. The STITCH database (http://stitch.embl.de/ (accessed on 1 June 2023)) [22] was used to identify the potential target proteins of drug-like compounds. Interactions for which the predictions were highly confident (combined score > 0.7) were chosen from the predicted interactions between compounds and targets [23]. The herb–compound–target network was built by connecting herb nodes, compound nodes, and target nodes, using the drug-like compounds of AR-specific herbs and their potential targets. For visualization, an open-source visualization and exploration software for graphs and networks, Gephi, was used [24].

### 2.4. Identification of Herb Clusters

To identify the subgroups of AR-specific herbs, we calculated the cosine similarity between every pair of herbs using their compounds and target vectors. The cosine similarity is a measurement that quantifies the similarity between two vectors. The cosine similarity is defined as the cosine of the angle between the two vectors, that is, the dot product of the vectors divided by the product of their lengths. Clustering analysis was conducted using Ward’s hierarchical agglomerative clustering method [25] in scikit-learn, and herb clusters were detected by cutting the dendrogram at the second level.

### 2.5. PPI Network and Gene Ontology (GO) Enrichment Analysis

To identify the characteristics of AR-specific herbs at the protein level, we constructed a PPI network using the predicted target proteins of the AR-specific herbs. The PPI network is a unipartite network in which nodes are defined as target proteins of AR-specific herbs and the edges are defined as PPIs. The Search Tool for Retrieval of Interaction Genes (STRING; https://string-db.org (accessed on 1 June 2023)) database, which integrates both known and predicted PPIs, was used to predict the functional interactions of proteins. The constructed network includes 28,898 PPIs, connecting 1509 unique proteins. The Louvain method for community detection was applied to extract the modules from a large PPI network. The Louvain algorithm is a hierarchical clustering algorithm that recursively merges communities into a single node and executes modularity clustering on condensed graphs [26]. GO enrichment analyses were conducted using Enrichr (http://amp.pharm.mssm.edu/Enrichr/ (accessed on 1 June 2023)) for modules obtained from the PPI network. To explore the role of each module, the top 10 GO enrichment terms were identified using the GO biological process 2021 gene-set library.

## 3. Results

### 3.1. Identification of AR-Specific Herbs

Among the numerous TAM herbal prescriptions used for AR, 12 representative prescriptions were selected by considering the AR clinical practice guidelines and the number of AR prescriptions in South Korea (Table S1). Among the 47 herbs included in the representative prescriptions for AR, 14 AR-specific herbs were selected by calculating the TF-IDF, which considers both the frequency of herbs within AR prescriptions and their rarity across all prescriptions. These herbs are likely to have specific therapeutic effects on AR, as they are more commonly used in AR prescriptions compared to other herbal prescriptions.

The 14 AR-specific herbs were ephedra intermedia, pueraria lobata, poria cocos, pinellia ternata, glycyrrhiza glabra, asiasarum sieboldii, cinnamomum cassia, bupleurum falcatum, platycodon grandiflorum, astragalus membranaceus, angelica decursiva, aralia continentalis, panax ginseng, and Saposhnikovia divaricata (poria cocos and astragalus membranaceus are synonym of wolfiporia extensa and astragalus mongholicus, respectively; Table 1).

### 3.2. AR-Related Genes and Enriched Pathways of AR-Specific Herbs

**Figure 1:**
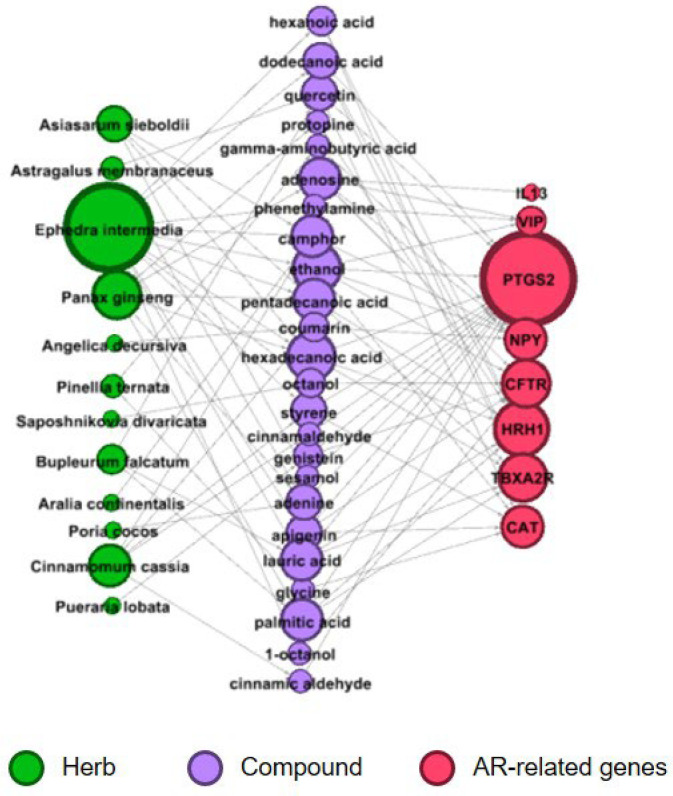
Representative herb–compound–target network for AR-related genes.

To infer the relationships between potential targets of AR-specific herbs and known AR-related genes, a disease-related herb–compound–target network was constructed (Figure 1). AR-related genes were retrieved from the CTD and DisGeNET databases (see the Section 2.2). Twelve out of the 14 AR-specific herbs were found to have targets overlapping with AR-related genes (Table 2). Among AR-specific herbs, ephedra intermedia had the highest number of AR-related genes, followed by panax ginseng, cinnamomum cassia, and asiasarum sieboldii (green circles in Figure 1). Among the overlapping AR-related genes, prostaglandin-endoperoxide synthase 2 (PTSG2) has the most interactions with AR-specific herbs, followed by histamine receptor H1 (HRH1), thromboxane A2 receptor (TBXA2R), and CF transmembrane conductance regulator (CFTR) (red circles in Figure 1).

Green, purple, and light red nodes represent herbs, bioactive compounds, and AR-related genes, respectively. AR-related genes were retrieved from the Comparative Toxicogenomics Database (CTD) and DisGeNet for rhinitis and allergic rhinitis. By comparing the predicted gene targets of the AR-specific herbs with the AR-related genes, overlapping genes and related compounds and herbs were visualized. The size of the nodes represents the degree of the network. AR = allergic rhinitis.

We then investigated which AR-related signaling pathways were associated with the targets of AR-specific herbs. We identified 17 AR-related pathways that consisted of one pathway in the KEGG pathway database and 16 AR-related pathways manually curated from previously published articles [27,28,29,30,31,32,33,34,35,36,37,38] (Table 3). In summary, AR-specific herbs affect AR through a wide range of pathways, including the regulation of cytokine and chemokine expression, cholinergic response, innate immunity, antigen processing and presentation, TRP ion channels, mast cell activation, goblet cell hyperplasia, T and B cells, and promotion of vascular permeability.

**Figure 2:**
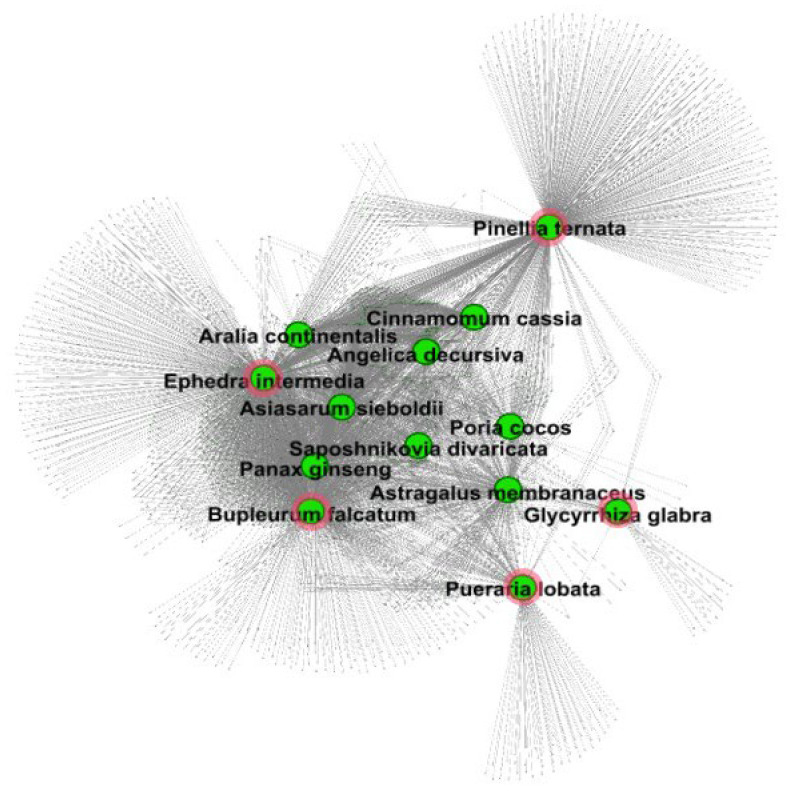
Herb–target network for AR-specific herbs. The green nodes represent the herbs and ends of the arrowheads for the predicted targets. As a result of filtering the compounds by the quantitative estimate of drug-likeness (QED) standard (QED > 0.35), platycodon grandiflorum was excluded because no active compound was included.

For a more comprehensive understanding of the anti-AR mechanisms of AR-specific herbs, we investigated their predicted targets. First, we constructed an herb–target network consisting of 14 herb nodes and 1975 predicted targets (Figure 2). We found that while most herbs shared targets that tended to congregate in the center, pinellia ternata, ephedra intermedia, bupleurum falcatum, pueraria lobata, and glycyrrhiza glabra had unique targets distinct from the shared targets in the center.

### 3.3. Investigation of Groups of AR-Specific Herbs Using PPI Network Analysis

**Figure 3:**
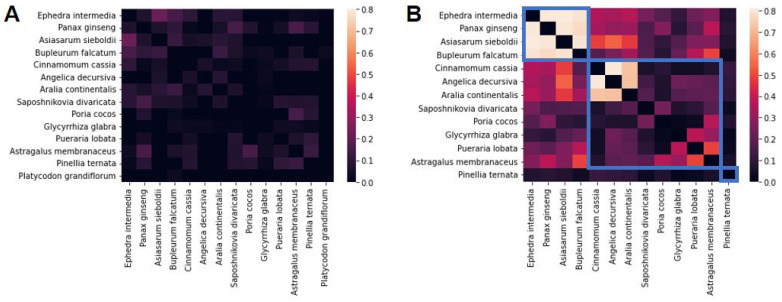
Herb clusters of the AR-specific herbs. (**A**) Cosine similarity of AR-specific herbs using compound information. (**B**) Cosine similarity of AR-specific herbs using target information. Three blue boxes indicate herb groups obtained from the hierarchical clustering analysis.

To identify the groups of AR-specific herbs, we conducted clustering analysis by calculating the cosine similarity between every pair of AR-specific herbs using their compound and target information. At the compound level, relatively low similarity was observed (average cosine similarity < 0.2; Figure 3A). At the target level, higher similarity was observed, including one with similarity > 0.8 (Figure 3B). As a result of hierarchical clustering analysis conducted on target information of AR-specific herbs, three subgroups were identified (blue boxes in Figure 3B). Group 1 included ephedra intermedia, panax ginseng, asiasarum sieboldii, and bupleurum falcatum; group 3 included only one herb, pinellia ternata; and group 2 included the remaining eight AR-specific herbs.

**Figure 4:**
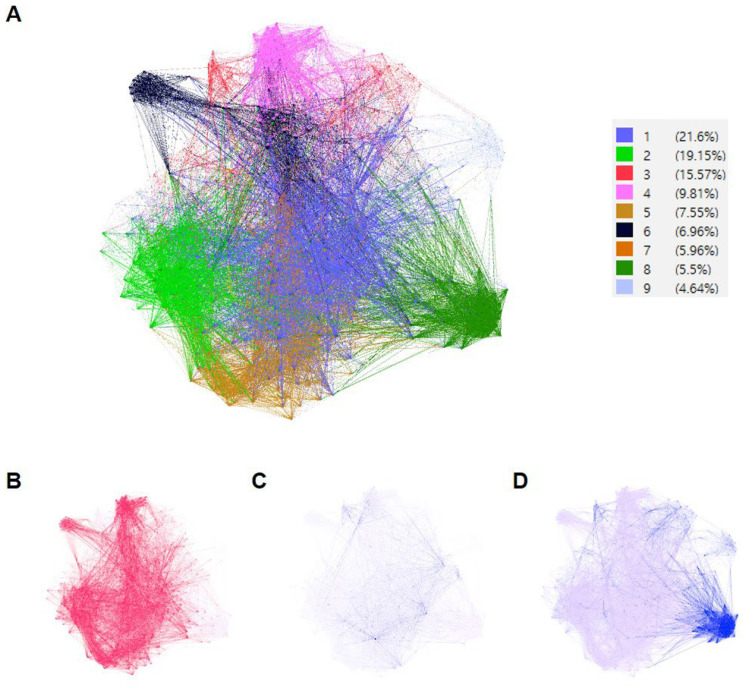
Protein–protein interaction (PPI) network analysis of AR-related herb clusters. (**A**) The PPI network was built with the targets of the AR-specific herbs. After constructing the PPI network, the modules were classified using the Louvain method. The percentage occupied by each color-coded module is shown in the legend. (**B**) The PPI network of group 1 herbs (ephedra intermedia, panax ginseng, asiasarum sieboldii, and bupleurum falcatum) is shown in red. (**C**) The PPI network of group 2 herbs (cinnamomum cassia, angelica decursiva, Aralia continentalis, pueraria lobata, poria cocos, glycyrrhiza glabra, astragalus membranaceus, saposhnikovia divaricata) is shown in dark blue. (**D**) The PPI network of group 3 herb (pinellia ternata) is shown in blue. AR = allergic rhinitis.

To identify the characteristics of the herb groups at the protein level, we constructed a PPI network using the predicted targets of all AR-specific herbs. Using the Louvain method for community detection, nine modules were observed in the PPI network (Figure 4A). The PPI network of group 1 herbs included modules 1, 2, 4, 5, 6, and 7 (Figure 4B); group 2 herbs included modules 1, 3, 5, and 7 (Figure 4C); and group 3 herbs included modules 3, 8, and 9 (Figure 4D). The PPI network of group 1 herbs covered most of the interactions of the network. The PPI network of the group 3 herb comprehensively covered the rest of the interactions, that is, an independent area that did not overlap with group 1, showing a tendency of agglomeration in the right bottom. The PPI network of group 2 herbs occupied a large area, partially overlapping with the interactions of group 1 and group 3 herbs. The color density of groups 1 and 3 was much darker than that of group 2, indicating that groups 1 and 3 had many more PPI interactions.

The top 10 GO biological process enrichment terms for each group are listed (Table 4). Although AR-specific herbs shared some common GOs, interestingly, each group showed distinct characteristics. Herbs from groups 1 and 2 were both highly related to GO terms such as ‘inflammatory response’, ‘cellular response to cytokine stimulus’, and ‘cytokine-mediated signaling pathway’, while the overlapping gene target lists were greater in group 1 than in group 2 (79/230, 94/482, and 104/621 in group 1; 55/230, 66/482, and 76/621 in group 2). Group 1 herbs were highly related to the ‘regulation of inflammatory response to antigenic stimulus’, while group 2 herbs were related to ‘detection of chemical stimuli involved in sensory perception of bitter taste’. Group 3 herbs were distinctly related to ‘antigen processing and presentation of exogeneous peptide antigen via MHC class I, TAP-dependent’ and ‘NIK/NF-kappaB signaling’ (overlap: 36/74, adjusted *p*-value: 1.18 × 10^−32^, combined score: 2499.68).

## 4. Discussion

In this study, to investigate the mechanisms of medicinal herbs specifically prescribed for AR in TAM, we identified 14 AR-specific herbs using the TF-IDF, which considers both the frequency of herbs within AR prescriptions and their rarity across all prescriptions. We analyzed whether AR-specific herbs are highly related to previously known AR-related genes and AR-related pathways. AR-specific herbs were found to target several genes related to inflammation and AR pathogenesis, such as PTGS2, HRH1, and TBXA2R. Pathway analysis revealed that AR-specific herbs were associated with multiple AR-related pathways, including cytokine signaling, immune response, and allergic inflammation. Furthermore, we identified the potential efficacy and mechanisms of AR-specific herb subgroups based on the PPI network related to these herbs. Clustering analysis based on target similarity identified three distinct subgroups of AR-specific herbs, each with potentially unique mechanisms of action. PPI network analysis showed that the herb subgroups targeted different sets of proteins and pathways, with some overlaps between groups. Group 1 herbs were associated with the regulation of inflammatory responses to antigenic stimuli, while Group 2 herbs were related to the detection of chemical stimuli involved in the sensory perception of bitter taste. Group 3 herbs were distinctly associated with antigen processing and presentation and NIK/NF-kappa B signaling. These findings suggest that the subgroups of AR-specific herbs may work synergistically to address different aspects of AR pathophysiology, providing a multi-targeted approach to the management of AR.

There are several mechanisms underlying AR, the most well-known of which is allergic sensitization. In AR, the initial allergen exposure and sensitization involves antigen-presenting cells at the mucosal site, leading to the activation of Th2 cells. Antigens trigger immunoglobulin E to bind to high-affinity receptors on the surface of mast cells, and diverse mediators of hypersensitivity, such as histamine, leukotrienes, and cytokines, are subsequently released [39]. Histamine, which is converted from histidine, mediates allergy and inflammation by activating the histamine receptors. The 5-lipooxygenase (LO) and prostaglandin E2 (PGE2) synthesis and signaling pathways are also related to AR. Several immune response pathways have also been identified.

Herbs in TAM prescriptions act on AR with multi-targets, derived from multiple components. This allows AR prescriptions to treat AR more effectively than synthetic drugs and has fewer side effects [40,41]. In previous research, many herbs and herbal decoctions showed efficacies on AR. Lim et al. [42] reviewed the efficacy and mechanisms of natural substances in AR therapies by examining data from 57 previous studies. Many herbs and herbal decoctions have shown efficacy in AR by regulating Th2 cytokines (interleukin (IL)-4, IL-10, and IL-13), interferon-gamma (IFN-γ), tumor necrosis factor-alpha (TNF-α), and phospho-ERK1/2 (p-ERK1/2), downregulating histamine receptors, and controlling the LO pathway, cyclooxygenase 2 (COX-2), LTC4, and PGE2. However, a systematic approach to understand the overall mechanisms of AR-specific herbs has not yet been reported.

In the current study, we identified AR-specific herbs using the TF-IDF method and investigated their potential effects and mechanisms in treating allergic rhinitis using a network pharmacological approach. Interestingly, there have been numerous studies exploring the efficacy and mechanisms of these selected AR-specific herbs in the context of allergic rhinitis.

Ephedra intermedia is widely used as a main herb in traditional medicine for treating various allergic diseases. Extensively researched for its adrenergic and anti-inflammatory properties, it serves as a notable source of ephedrine alkaloids. Ephedra protected mice from endotoxin damage by blocking the activation of Toll-like receptor 2 (TLR2), thereby reducing the expression of inflammatory factors in macrophages. The administration of ephedrine can produce an anti-inflammatory impact by suppressing the generation of stimulated inflammatory factors (like TNF-α) via the PI3K/Akt and PGN routes [43,44].

Pueraria lobata has been elucidated for its anti-inflammatory and anti-allergic effects in recent research, particularly demonstrating notable efficacy against pulmonary diseases and allergic asthma [45]. A study has demonstrated that puerarin has been observed to inhibit the activation of the NF-kappa B pathway and the production of TNF-α in peripheral blood mononuclear cells of asthmatic patients, suggesting its potential protective role against allergic diseases [46]. Additionally, puerarin can inhibit T lymphocyte action. A recent study showed that it restores the Th1/Th2 balance by facilitating OVA-induced immune shift from Th1 to Th2 cells during delayed-type hypersensitivity [46].

Pinellia ternata, extracted as a common herb for AR in this study, is recognized as one of the most representative herbal medicines in traditional medicine for eliminating phlegm (痰). Pharmacologically, it is well-documented for its anti-cough, expectorant, and anti-asthmatic effects rather than its properties for treating allergic rhinitis [47,48]. It has been identified in our study’s PPI network as possessing distinct therapeutic scope. The fact that our findings align with the results of these previous studies supports the validity and relevance of our research. The convergence of evidence from both our network pharmacological analysis and prior experimental studies on the anti-AR effects of these herbs strengthens the case for their potential use in the treatment of allergic rhinitis.

In the analysis of AR-related genes (Figure 1), PTSG2 showed the strongest association with AR-specific herbs, followed by HRH1, TBXA2R, and CFTR. This result shows how AR-specific herbs act on AR symptoms. PTSG2, also known as COX-2, is responsible for the production of large amounts of proinflammatory prostaglandins at the inflammatory site [49]. HRH1 contributes to the pathophysiology of AR by mediating the actions of histamine [50]. TBXA2R is known to stimulate vascular endothelial cell pro-inflammatory responses [51]. CFTR, as a chloride channel, controls ion and water secretion and absorption in epithelial tissues, thereby affecting mucus production [52].

We also clustered AR-specific herbs into three groups at the target level and constructed a PPI network using all AR-specific herbs and each herb group (Figure 3, 4). As shown in Table S1, representative prescriptions of AR included herbs from all three groups, indicating that herbs of various characteristics are gathered in one prescription. Considering the mechanisms of the three herb groups, we found that these groups have parallels in the TAM prescription design principle, named ‘Kun-Shin-Choa-Sa’ [10]. In the Kun-Shin-Choa-Sa principle, the kun (king; major component) indicates a major herb conveying the major drug efficacy and/or the greatest dose, and is supported by three different types of medicines: Shin (minister; complementary component) for enhancing and/or complementing the efficacy of the Kun, Choa (assistant; neutralizing component) for reducing side effects caused by the Kun and reducing the minor symptoms accompanying major symptoms, and Sa (ambassador; delivery/retaining component) to facilitate the delivery of the Kun to the target site, and retaining the Kun for prolonged availability in the cells.

Interestingly, group 1 herbs were Kun or Shin herbs commonly used for AR (Table S1). As shown in Figure 4, the PPI network of group 2 mostly overlapped with that of group 1 and was distributed slightly more widely, indicating that group 2 herbs support the effects of group 1 herbs that have stronger efficacy. Only pinellia ternata was clustered in group 3, indicating that it had unique targets separate from other herbs. Consistent with this result, pinellia ternata also had its own area represented as modules 3, 8, and 9 in the PPI network. We also found that pinellia ternata mostly belonged to Sa (Table S1). Altogether, these results indicate the reasons for pinellia ternata being solely clustered as group 3. The results of clustering analysis and subsequent PPI network analysis show how AR-specific herbs work independently and cooperatively at the same time, acting in a synergistic manner, including strengthening efficacy, reducing side effects, facilitating action, and pharmacokinetic potentiation [10].

We also investigated group-specific mechanisms of AR. As a result of enrichment analysis (Table 4), group 1 herbs showed high relevance with ‘regulation of inflammatory response to antigenic stimulus’; group 2 herbs with ‘detection of chemical stimulus involved in sensory perception of bitter taste’; group 3 herbs with ‘antigen processing and presentation of exogeneous peptide antigen via MHC class I, TAP-dependent’ and ‘NIK/NF-kappa B signaling’. In addition, both group 1 and 2 herbs were highly related to ‘cytokine-mediated signaling pathway’. These biological processes have been reported to be highly associated with AR, especially NF-kappa B signaling. It has been reported that local inhibition of NF-kappa B suppressed allergic response, suggesting its therapeutic impact in the treatment of AR [36]. In addition, a high relevance has been reported between bitter taste receptors and vasoconstriction in AR. Bitter substance-mediated activation of the bitter taste receptor, TAS2R, in the nasal mucosa could lead to vasoconstrictor responses [53,54]. Group 2 herbs may promote vasoconstriction by acting on bitter taste receptors.

In this study, we interpreted how many herbs act harmoniously in each AR prescription from network biology approach and TAM theory. Since the human body is a complex interacting system of many networks, multitarget approaches are of interest as potential means of treating complex diseases [10]. Due to multicomponent and multitargets base, understanding of the mechanisms of action of TAM prescriptions needs systems-based network pharmacological approach. Simultaneously, our study has several limitations that should be acknowledged. Firstly, the network pharmacological approach relies on existing databases and computational predictions, which may not fully capture the complexity of the biological systems involved in AR. To address this limitation, we have used well-established and widely used databases, such as STITCH and KEGG, and applied stringent criteria for selecting high-confidence interactions. Furthermore, we have integrated information from multiple sources, such as using experimentally validated AR-related genes from CTD and DisGeNet, and employed various computational methods to enhance the robustness of our findings. Another limitation is that our study focuses on a specific set of AR-specific herbs identified through the TF-IDF method, which may not represent the full spectrum of herbs used in traditional Asian medicine for treating AR. However, we believe that our approach of selecting the most frequently mentioned herbs in the context of AR provides a valuable starting point for understanding the key players and mechanisms involved. To further validate our findings and address the limitations of the computational approach, we suggest that future studies incorporate experimental validation of the predicted targets and interactions, as well as the mechanisms of action of the identified AR-specific herbs and their combinations in the treatment of allergic rhinitis.

## 5. Conclusions

In this study, we attempted to decipher how multiple herbs act harmoniously in each AR prescription, using a network pharmacology approach. We identified AR-specific herbs, which allowed for a more precise analysis of the herbs used in AR prescriptions. We confirmed that the targets of AR-specific herbs and known AR-related genes largely overlap. Subgroups of AR-specific herbs with distinct mechanisms of action were identified. We were able to interpret the TAM principle behind the configuration of herbal prescriptions for AR.

## Figures and Tables

**Table 1:** Herbal name index.

Scientific Name	Latin Name	Chinese Name
*Ephedra intermedia*	Ephedra Herba	Ma Huang (麻黃)
*Pueraria lobata*	Puerariae Radix	Ge Gen (葛根)
*Poria cocos*	Poria Sclerotium	Fu Ling (茯苓)
*Pinellia ternata*	Pinelliae Tuber	Ban Xia (半夏)
*Glycyrrhiza glabra*	Glycyrrhizae Radix et Rhizoma	Gan Cao (甘草)
*Asiasarum sieboldii*	Asiasari Radix et Rhizoma	Xi Xin (細辛)
*Cinnamomum cassia*	Cinnamomi Ramulus	Gui Zhi (肉桂)
*Bupleurum falcatum*	Bupleuri Radix	Chai Hu (柴胡)
*Platycodon grandiflorum*	Platycodonis Radix	Jie Geng (桔梗)
*Astragalus membranaceus*	Astragali Radix	Huang Qi (黃芪)
*Angelica decursiva*	Peucedani Radix	Qian Hu (前胡)
*Angelica pubescens*	Angelica Pubescens Radix	Du Huo (獨活)
*Panax ginseng*	Ginseng Radix	Ren Shen (人蔘)
*Saposhnikovia divaricata*	Saposhnikoviae Radix	Fang Feng (防風)

**Table 2:** Herb–compound–target network of AR-specific herbs.

Herb name	Number of H-C Interactions	Number of C-T Interactions	Number of Overlapped Genes (Interactions) Between Targets of AR-Specific Herbs and AR-Related Genes	Overlapped Gene Name (Number of C-T Interactions)
*Ephedra intermedia*	106	2021	7(27)	CAT(3), PTGS2(6), HRH1(5), TBXA2R(5), VIP(2), NPY(2), CFTR(4)
*Pueraria lobata*	16	207	2	PTGS2, CFTR
*Poria cocos*	13	122	1	PTGS2
*Pinellia ternata*	25	1553	2	CAT, NPY
*Glycyrrhiza glabra*	82	74	0	-
*Asiasarum sieboldii*	73	914	4(7)	PTGS2(2), HRH1(2), TBXA2R(2), NPY
*Cinnamomum cassia*	18	448	3(5)	CAT, PTGS2(2), NPY(2)
*Bupleurum falcatum*	56	948	4(8)	CAT, PTGS2(3), HRH1(2), TBXA2R(2)
*Platycodon grandiflorum*	6	0	0	-
*Astragalus membranaceus*	20	405	2(3)	CAT, PTGS2(2)
*Angelica decursiva*	16	263	1	NPY
*Aralia continentalis*	79	311	1	NPY
*Panax ginseng*	88	1707	7(14)	PTGS2(3), IL13, HRH1(4), TBXA2R(3), VIP, NPY, CFTR
*Saposhnikovia divaricata*	41	81	1	CFTR

H, herb; C, compound; T, target.

**Table 3:** The result of pathway enrichment analysis.

Pathway			Overlap	Adjusted *p*-Value	Combined Score
	KEGG pathway database				
		Cytokine–cytokine receptor interaction	78/295	6.42 × 10^−16^	121.53
	Previous research				
		Chemokine signaling pathway [29]	110/192	1.47 × 10^−58^	1771.89
		Cholinergic synapse [32]	77/113	3.55 × 10^−49^	2331.14
		Inflammatory mediator regulation of TRP channels [31]	57/98	2.95 × 10^−31^	947.24
		TNF signaling pathway [27]	49/112	3.80 × 10^−20^	334.19
		VEGF signaling pathway [30]	34/59	4.49 × 10^−19^	548.67
		IL-17 signaling pathway [33]	43/94	9.56 × 10^−19^	335.07
		T cell receptor signaling pathway [33]	40/104	1.86 × 10^−14^	188.84
		Toll-like receptor signaling pathway [34]	38/104	5.26 × 10^−13^	155.29
		Histidine metabolism [35]	16/22	6.86 × 10^−12^	648.97
		Th17 cell differentiation [33]	37/107	7.01 × 10^−12^	129.43
		Antigen processing and presentation [33]	29/78	1.90 × 10^−10^	126.10
		B cell receptor signaling pathway [33]	29/81	5.16 × 10^−10^	113.37
		Fc epsilon RI signaling pathway [28,38]	26/68	7.89 × 10^−10^	123.12
		Th1 and Th2 cell differentiation [33]	30/92	3.25 × 10^−09^	89.90
		NF-kappa B signaling pathway [36]	32/104	4.80 × 10^−09^	80.94
		TGF-beta signaling pathway [37]	17/94	0.012365	9.30

**Table 4:** Top 10 of GO biological process terms for each group sorted by adjusted *p*-value.

GO Term	Overlap	Adjusted *p*-Value	Combined Score
Group 1			
adenylate cyclase-modulating G protein-coupled receptor signaling pathway (GO:0007188)	83/165	1.19 × 10^−55^	2472.19
positive regulation of cytosolic calcium ion concentration (GO:0007204)	71/147	6.81 × 10^−46^	1868.84
retinoid metabolic process (GO:0001523)	56/92	2.22 × 10^−43^	2904.11
**negative regulation of inflammatory response (GO:0050728)**	81/212	2.67 × 10^−43^	1170.88
**regulation of inflammatory response to antigenic stimulus (GO:0002861)**	63/137	3.44 × 10^−39^	1442.96
regulation of cytosolic calcium ion concentration (GO:0051480)	65/148	5.14 × 10^−39^	1321.08
**inflammatory response (GO:0006954)**	79/230	1.86 × 10^−38^	877.027
**negative regulation of inflammatory response to antigenic stimulus (GO:0002862)**	62/136	1.86 × 10^−38^	1386.64
adenylate cyclase-activating G protein-coupled receptor signaling pathway (GO:0007189)	57/118	4.97 × 10^−37^	1484
phospholipase C-activating G protein-coupled receptor signaling pathway (GO:0007200)	48/81	1.30 × 10^−36^	2266.77
Group 2			
**inflammatory response (GO:0006954)**	55/230	1.19 × 10^−30^	867.789
adenylate cyclase-inhibiting G protein-coupled receptor signaling pathway (GO:0007193)	32/60	4.54 × 10^−30^	2968.78
**detection of chemical stimulus involved in sensory perception of bitter taste (GO:0001580)**	25/40	1.17 × 10^−25^	3676.68
**sensory perception of bitter taste (GO:0050913)**	25/41	2.19 × 10^−25^	3397.9
**detection of chemical stimulus involved in sensory perception of taste (GO:0050912)**	25/44	2.20 × 10^−24^	2747.01
**cytokine-mediated signaling pathway (GO:0019221)**	76/621	1.54 × 10^−23^	300.39
**cellular response to cytokine stimulus (GO:0071345)**	66/482	5.56 × 10^−23^	329.317
adenylate cyclase-modulating G protein-coupled receptor signaling pathway (GO:0007188)	40/165	9.92 × 10^−23^	634.668
positive regulation of cytosolic calcium ion concentration (GO:0007204)	37/147	1.38 × 10^−21^	631.861
estrogen metabolic process (GO:0008210)	20/30	1.46 × 10^−21^	3647.25
Group 3			
regulation of cellular amino acid metabolic process (GO:0006521)	37/54	2.92 × 10^−40^	7248.12
regulation of cellular amine metabolic process (GO:0033238)	36/51	3.30 × 10^−40^	7906.43
**antigen processing and presentation of exogenous peptide antigen via MHC class I, TAP-dependent (GO:0002479)**	41/73	3.30 × 10^−40^	4225.02
**antigen processing and presentation of exogenous peptide antigen via MHC class I (GO:0042590)**	42/78	3.30 × 10^−40^	3849.61
pre-replicative complex assembly (GO:0036388)	39/64	3.30 × 10^−40^	5120.1
negative regulation of cell cycle G2/M phase transition (GO:1902750)	36/57	6.80 × 10^−38^	5281.22
regulation of transcription from RNA polymerase II promoter in response to hypoxia (GO:0061418)	39/75	1.40 × 10^−36^	3236.09
anaphase-promoting complex-dependent catabolic process (GO:0031145)	40/84	1.32 × 10^−35^	2646.42
regulation of cellular ketone metabolic process (GO:0010565)	36/64	1.95 × 10^−35^	3697.92
regulation of transcription from RNA polymerase II promoter in response to stress (GO:0043618)	40/87	6.33 × 10^−35^	2425.64

Bold text indicates GO biological processes related to AR.

## Data Availability

Data will be made available on request.

## Supplementary Materials

The following supporting information can be downloaded at: https://www.mdpi.com/article/10.3390/life14050553/s1, Table S1: Representative prescriptions for allergic rhinitis analyzed in this study; Table S2: Calculation process of disease specificity; Table S3: Compound-target interactions of overlapped genes between targets of AR-specific herbs and AR-related genes.
