# Supplementary material for: Deciphering the Systemic Impact of Herbal Medicines on Allergic Rhinitis: A Network Pharmacological Approach
Source: Life (Basel). 2024 Apr 25;14(5):553. doi: 10.3390/life14050553 (PMC11122645; doi:10.3390/life14050553)
Supplement: Supplementary file 1 [file life-14-00553-s001.zip › life-2948797-supplementary.pdf]

Supplementary Table S1. Representative prescriptions for allergic rhinitis analyzed in this study.

Prescription	Kun	Shin	Choa	Sa
Socheongryong-tang[13]	Ephedra intermedia<br>Cinnamomum cassia	Zingiber officinale Roscoe<br>Asiasarum sieboldii	Schisandra chinensis Baillon<br>Paeonia lactiflora Pallas<br>Pinellia ternata	Glycyrrhiza glabra
Okbyungpoong-san[14]	Astragalus membranaceus	Atractylodes macrocephala Koidzumi	Saposhnikovia divaricata	
Bojungikgi-tang[13]	Astragalus membranaceus	Panax ginseng<br>Glycyrrhiza glabra<br>Atractylodes macrocephala Koidzumi	Angelica gigas Nakai<br>Citrus unshiu Markovich<br>Cimicifuga dahurica Maximowicz<br>Bupleurum falcatum	Glycyrrhiza glabra
Mahwangbujaseshin-tang[14]	Ephedra intermedia<br>Aconitum carmichaelii	Asiasarum sieboldii		
Hyongeyonggyo-tang	Schizonepeta tenuifolia Briquet<br>Bupleurum falcatum ~~Linné~~<br>Ligusticum chuanxiong<br>Angelica gigas Nakai<br>Rehmannia glutinosa Liboschitz ex Steudel<br>Paeonia lactiflora Pallas<br>Angelica dahurica<br>Saposhnikovia divaricate<br>Mentha arvensis Linné var. piperascens Malinvaud ex Holmes<br>Gardenia jasminoides Ellis<br>Scutellaria baicalensis Georgi<br>Platycodon grandiflorum A. De Candolle<br>Forsythia suspensa Vahl<br>Glycyrrhiza glabra			
Samsoeum[13]	Perilla frutescens Britton<br>Pueraria lobata	Angelica decursiva<br>Pinellia ternata<br>Platycodon grandiflorum A. De Candolle<br>Citrus unshiu Markovich<br>Citrus aurantium	Panax ginseng<br>Poria cocos<br>Aucklandia lappa Decne.	Glycyrrhiza glabra
Galgeun-tang[15]	Pueraria lobata	Ephedra intermedia	Cinnamomum cassia	Glycyrrhiza glabra

			Paeonia lactiflora Pallas	Zingiber officinale Roscoe<br>Zizyphus jujuba Miller
Yeotaectonggi-tang[16]	Astragalus membranaceus	Atractylodes lancea DC.<br>Ostericum koreanum<br>Aralia continentalis<br>Saposhnikovia divaricata<br>Cimicifuga dahurica Maximowicz<br>Pueraria lobata<br>Glycyrrhiza glabra<br>Ephedra intermedia<br>Rubia akane Nakai<br>Angelica dahurica<br>Zingiber officinale Roscoe<br>Zizyphus jujuba Miller<br>Allium fistulosum Linné		
Younggamgangmisinhayin-tang[17]	Poria cocos	Zingiber officinale Roscoe<br>Asiasarum sieboldii	Schisandra chinensis Baillon<br>Prunus armeniaca L. var. ansu Maxim.	Glycyrrhiza glabra<br>Pinellia ternata
Insampaedok-san	Angelica decursiva<br>Aralia continentalis<br>Bupleurum falcatum<br>Citrus aurantium L.<br>Cnidium officinale Makino<br>Forsythia viridissima Lindl.<br>Glycyrrhiza uralensis Fisch.<br>Lonicera japonica Thunb.<br>Mentha arvensis L.<br>Ostericum koreanum Kitag.<br>Panax ginseng C. A. Mey.<br>Platycodon grandiflorus A. DC.<br>Poria cocos Wolf<br>Saposhnikovia divaricata Schischk.<br>Schizonepeta tenuifolia Briq.<br>Zingiber officinale Roscoe			
Yukmijihwang-tang[13]	Rehmannia glutinosa Liboschitz ex Steudel	Cornus officinalis Siebold et Zuccarini<br>Dioscorea batatas Decaisne	Alisma orientale (Sam.) Juz.<br>Paeonia suffruticosa Andrews<br>Poria cocos	
Maekmundong-tang[13]	Ophiopogon japonicus Ker-Gawler	Panax ginseng<br>Glycyrrhiza glabra<br>Oryza sativa Linné<br>Zizyphus jujuba Miller	Pinellia ternata	Glycyrrhiza glabra

Supplementary Table S2. Calculation process of disease specificity

Herb	Frequency in TCMID	TF	Ln(IDF)	TF x LN(IDF) x 100
Ephedra intermedia	1753	0.057143	3.287307	18.78461266
Pueraria lobata	1240	0.042857	3.633524	15.57224761
Poria cocos	3739	0.057143	2.529818	14.45610072
Pinellia ternata	4086	0.057143	2.441069	13.94896757
Glycyrrhiza glabra	11659	0.1	1.392557	13.92557407
Asiasarum sieboldii	2044	0.042857	3.133727	13.43025921
Cinnamomum cassia Presl	633	0.028571	4.305921	12.30263051
Bupleurum falcatum	2712	0.042857	2.850949	12.21835479
Platycodon grandiflorum	2775	0.042857	2.827985	12.11993603
Astragalus membranaceus	2980	0.042857	2.756713	11.81448223
Angelica decursiva	1015	0.028571	3.833747	10.95356345
Aralia continentalis	1137	0.028571	3.720243	10.62926459
Panax ginseng	7553	0.057143	1.826691	10.43823421
Saposhnikovia divaricata	4181	0.042857	2.418085	10.36322301
Schisandra chinensis Baillon	1821	0.028571	3.24925	9.283571487
Atractylodes macrocephala Koidzumi	5469	0.042857	2.14954	9.212314444
Cimicifuga dahurica Maximowicz	2066	0.028571	3.123021	8.92291843
Ostericum koreanum	2204	0.028571	3.058362	8.738176943
Angelica dahurica	2325	0.028571	3.004916	8.585473663
Zingiber officinale Roscoe	2847	0.028571	2.80237	8.006771466
Citrus aurantium	2858	0.028571	2.798514	7.995753548
Paeonia lactiflora Pallas	3228	0.028571	2.676773	7.64792306
Citrus unshiu Markovich	3829	0.028571	2.506032	7.160091854
Ligusticum chuanxiong	4140	0.028571	2.42794	6.936971524
Angelica gigas Nakai	8262	0.028571	1.736969	4.962768949

Supplementary Table S3. Compound-target interactions of overlapped genes between targets of AR-specific herbs and AR-related genes

Herb	Compound	Gene	Herb	Compound	Gene
Ephedra intermedia	apigenin	CAT	*Asiasarum sieboldii*	pentadecanoic acid	TBXA2R
	styrene	CAT		hexadecanoic acid	TBXA2R
	ethanol	CAT		camphor	NPY
	apigenin	PTGS2	*Cinnamomum cassia*	styrene	CAT
	styrene	PTGS2		styrene	PTGS2
	dodecanoic acid	PTGS2		cinnamaldehyde	PTGS2
	lauric acid	PTGS2		camphor	NPY
	hexadecanoic acid	PTGS2		coumarin	NPY
	ethanol	PTGS2	*Bupleurum falcatum*	quercetin	CAT
	hexanoic acid	HRH1		lauric acid	PTGS2
	pentadecanoic acid	HRH1		palmitic acid	PTGS2
	dodecanoic acid	HRH1		quercetin	PTGS2
	lauric acid	HRH1		lauric acid	HRH1
	hexadecanoic acid	HRH1		palmitic acid	HRH1
	hexanoic acid	TBXA2R		lauric acid	TBXA2R
	pentadecanoic acid	TBXA2R		palmitic acid	TBXA2R
	dodecanoic acid	TBXA2R	*Astragalus membranaceus*	quercetin	CAT
	lauric acid	TBXA2R		quercetin	PTGS2
	hexadecanoic acid	TBXA2R		adenine	PTGS2
	phenethylamine	VIP	*Angelica decursiva*	coumarin	NPY
	ethanol	VIP	*Aralia continentalis*	camphor	NPY
	camphor	NPY	*Panax ginseng*	palmitic acid	PTGS2
	ethanol	NPY		hexadecanoic acid	PTGS2
	apigenin	CFTR		adenine	PTGS2
	octanol	CFTR		adenosine	IL13
	1-octanol	CFTR		pentadecanoic acid	HRH1
	ethanol	CFTR		palmitic acid	HRH1
Pueraria lobata	genistein	PTGS2		hexadecanoic acid	HRH1
	genistein	CFTR		protopine	HRH1
Poria cocos	adenine	PTGS2		pentadecanoic acid	TBXA2R
Pinellia ternata	glycine	CAT		palmitic acid	TBXA2R
	gamma-aminobutyric acid	NPY		hexadecanoic acid	TBXA2R
Asiasarum sieboldii	hexadecanoic acid	PTGS2		adenosine	VIP
	sesamol	PTGS2		adenosine	NPY
	pentadecanoic acid	HRH1		adenosine	CFTR
	hexadecanoic acid	HRH1	*Saposhnikovia divaricata*	octanol	CFTR
